# Stormbow: A Cloud-Based Tool for Reads Mapping and Expression Quantification in Large-Scale RNA-Seq Studies

**DOI:** 10.1155/2013/481545

**Published:** 2013-09-11

**Authors:** Shanrong Zhao, Kurt Prenger, Lance Smith

**Affiliations:** ^1^Systems Pharmacology and Biomarkers, Janssen Research & Development, LLC, 3210 Merryfield Row, San Diego, CA 92121, USA; ^2^High Performance & Scientific Computing, Janssen Research & Development, LLC, 920 Route 202, Raritan, NJ 08869, USA; ^3^Translational Informatics IT, Janssen Research & Development, LLC, 3210 Merryfield Row, San Diego, CA 92121, USA

## Abstract

RNA-Seq is becoming a promising replacement to microarrays in transcriptome profiling and differential gene expression study. Technical improvements have decreased sequencing costs and, as a result, the size and number of RNA-Seq datasets have increased rapidly. However, the increasing volume of data from large-scale RNA-Seq studies poses a practical challenge for data analysis in a local environment. To meet this challenge, we developed Stormbow, a cloud-based software package, to process large volumes of RNA-Seq data in parallel. The performance of Stormbow has been tested by practically applying it to analyse 178 RNA-Seq samples in the cloud. In our test, it took 6 to 8 hours to process an RNA-Seq sample with 100 million reads, and the average cost was $3.50 per sample. Utilizing Amazon Web Services as the infrastructure for Stormbow allows us to easily scale up to handle large datasets with on-demand computational resources. Stormbow is a scalable, cost effective, and open-source based tool for large-scale RNA-Seq data analysis. Stormbow can be freely downloaded and can be used out of box to process Illumina RNA-Seq datasets.

## 1. Introduction

RNA-Seq is the direct sequencing of transcripts by high-throughput sequencing technology and can profile an entire transcriptome at single-base resolution whilst concurrently quantifying gene expression levels on a genome-wide scale [1–3]. RNA-Seq not only has considerable advantages for examining transcriptome fine structure—for example, in the detection of novel transcripts, allele-specific expression, and alternative splicing—but also provides a far more precise measurement of levels of transcripts than that of other methods [4, 5]. With no probes or primers to design, RNA-Seq delivers unbiased and unparalleled information about the transcriptome and gene expression. Early studies have demonstrated that RNA-Seq is very reliable in terms of technical reproducibility [6, 7]. Compared to microarray-based profiling, RNA-Seq can detect the expression of low abundance transcripts and the subtle change under different conditions; has a wider dynamic range; and avoids technical issues in microarray related to probe performance such as cross-hybridization, limited detection range of individual probes, and nonspecific hybridization [8, 9]. Currently, RNA-Seq is becoming an attractive approach in the profiling of gene expression and in evaluating differential expression [10–13].

Until recently, sequencing has primarily been carried out in large genome centers which have invested heavily in computational infrastructure that enables genomic sequence analysis [14, 15]. The recent advancements in sequencing technology have greatly decreased the sequencing costs and increased the size and number of datasets. As a consequence, larger amounts of sequence data are not only being produced at lower costs, but also being more often by small to midsize research groups. It is expected that clinical sequencing will become produced a part of the diagnostic routines shortly [16]. However, the enormous data from large-scale RNA-Seq studies poses a fundamental problem of data management and analysis in a local environment and requires increasingly complex computational methods [17]. Consequently, limited access to computational infrastructure and high-quality bioinformatics tools and the demand for personnel skilled in data analysis and interpretation remain a serious bottleneck for most researchers. 

In recent years, cloud computing has emerged as a viable option to quickly and easily acquire computational resources for large-scale data analysis [18–22]. Several cloud-based bioinformatics applications and resources have been developed specifically to address the challenges of working with the very large volumes of data generated by Next Generation Sequencing (NGS) technology, including CloudBurst [23], Crossbow [24], Myrna [25], CloVR [26], CloudAligner [27], and PeakRanger [28]. Cloud computing has created new possibilities to analyze NGS data at reasonable costs, especially for laboratories lacking a dedicated bioinformatics infrastructure. 

Differential analysis of RNA-Seq data consists of three key steps [10]: (i) reads mapping; (ii) expression quantification; and (iii) statistical testing to determine significantly differential expressed (DE) genes. For statistical tests, several R-based packages have recently been developed specifically for DE analysis in RNA-Seq experiments, including DESeq [29], edgeR [30], and baySeq [31]. As an input, these algorithms take count data in the form of a rectangular table of integer values. The table cell in the *i*th row and the *j*th column of the table corresponds to the number of sequence reads mapped to gene *i* in sample *j*. To obtain such a count table, we need to generate it from the raw sequence reads, which is done by steps (i) and (ii) that are mentioned above. Both steps are very computationally intensive [2], especially for RNA-Seq studies in which hundreds or even thousands of samples are sequenced. 

Stormbow, as described in this paper, is a cloud-based tool developed to meet the computational challenges in large-scale RNA-Seq data analysis and to significantly reduce the turnaround time in RNA-Seq data analysis. The tool simply takes raw sequence reads in FASTA format as inputs and generates a gene count table for large RNA-Seq studies in a very short time by processing individual RNA-Seq samples in parallel using the cloud. Stormbow is an extension of Rainbow [32], a software package that we developed for large-scale whole genome sequence data analysis in the cloud. Both Rainbow and Stormbow were developed to meet the computational challenges in large-scale genomic sequencing, with Rainbow for whole-genome sequencing while Stormbow for RNA-Seq. Stormbow can be freely downloaded from http://s3.amazonaws.com/jnj_stormbow/index.html.

## 2. Materials and Methods

**Figure 1:**
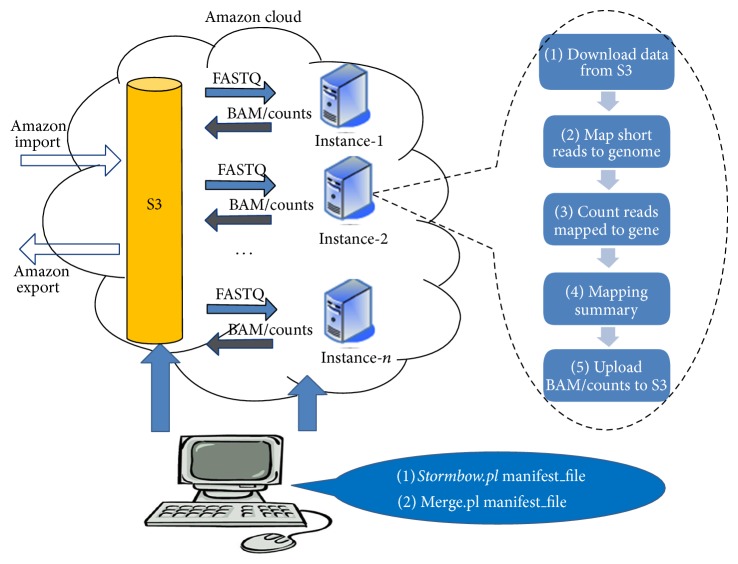
Stormbow in action. S3 centralizes data storage. The large volumes of data are imported into or exported from S3 through Amazon Import and Export services. Multiple EC2 instances are launched automatically from a local Linux workstation by the Perl script, *Stormbow.pl*. All EC2 instances fetch sequence data from S3 and upload result files to S3. The key steps and tasks performed by each EC2 instance are detailed in the right of the figure. The *Merge.pl* script combines the gene counts in each sample into a consolidated count table that may be used as input to differential analysis tools, such as DESeq and edgeR.

The architecture of Stormbow is shown in Figure 1. Amazon's Simple Storage Service (S3) [33] centralizes data storage. Stormbow comes with scripts that automate the Stormbow pipeline on Amazon's Elastic Compute Cloud (EC2) [34] utility computing service. The EC2 driver script can be run from any Linux computer with internet connection; however, all the computations are executed remotely by multiple EC2 instances in the cloud. Below, we describe the architectural design for Stormbow step by step.

### 2.1. The Algorithm for Mapping Reads

There are multiple open-source based aligners available to mapping reads to a reference genome. With Stormbow, we wrap the Omicsoft Sequence Aligner (OSA) [35], a fast and accurate alignment tool for RNA-Seq, and run it in Amazon's cloud environment. Benchmarked with existing methods, such as TopHat [36], OSA improves mapping speed 4–10*x* with better sensitivity and less false positives than similar tools. The detailed benchmarking results are reported at the OSA website [37]. OSA has built-in features to automatically trim those nucleotide bases with low-quality score at 3′-end when it maps sequence reads to a reference transcriptome and genome. For raw sequence data, no additional preprocessing is needed. 

### 2.2. Amazon's Import/Export Service and S3 for Data Storage

The raw sequence data for a large-scale RNA-Seq study, in which several hundred samples are sequenced, is typically multiple terabytes (TB) in size. For a deep RNA-Seq sample, the raw sequence data in FASTQ format ranges from 20 to 25 GB, and after reads mapping, the corresponding BAM file (Binary version of a Sequence Alignment Map) ranges from 8 to 10 GB. Assuming 200 samples, the input data will be 4 to 5 TB, with the output BAM files adding another 2 TB. For such large volumes of data, it is impractical to transfer them to and from Amazon via a typical network connection. The most efficient and reliable way is to move them through Amazon's Import/Export service. This service allows a user to ship multiple hard drives via FedEx to Amazon; after which, Amazon copies the data to S3 directly. The user has the option to encrypt the data on the hard drives to ensure security during transit. This process usually takes 2 to 3 days. When Stormbow runs, it can fetch the sequence data from S3 directly (see Figure 1). In the Stormbow pipeline, S3 centralizes data storage, including inputs, intermediate results, and outputs. After an EC2 instance finishes its computational tasks, it is instructed to terminate itself. After termination, all data in that instance is inaccessible. Therefore, the result files must be uploaded to S3 prior to instance termination. S3 provides virtually unlimited storage space for cloud computing, ensures high durability of its data, and allows for parallel I/O, which is why we chose S3 as the centralized data storage for the Stormbow pipeline.

### 2.3. The Compute Node Type and Configuration for RNA-Seq Data Analysis

Amazon offers different types of machines with varying disk sizes and CPU configurations. Users can select and configure these machines to stratify their computational needs. However, Amazon does not offer an analysis solution; it is the user's responsibility to properly utilize the resources to analyse data. After requested resources from Amazon become available, they must be configured with the necessary software, datasets must be downloaded, and then analysis can be run.

OSA requires at least 8 GB of memory to run. After benchmarking, the m1.xlarge EC2 instance type was chosen to run OSA. The m1.xlarge instance consists of a 64 bit platform, 15 GB memory, 4 virtual cores, 1,690 GB instance storage, and very high I/O performance. Since there are 4 cores, 4 parallel threads can be launched to map millions of short reads to the reference genome and gene model. After an m1.xlarge instance is requested, these required software packages are installed, including the following: (1) *zlib-devel*, a library for data compression and inflation; (2) *mono-2.10.8* [38] to provide a runtime environment in Linux for applications that require  .NET framework support; (3) OSA software; (4) hg19 (the human reference genome in its 19th version); (5) RefSeq gene model—both the reference genome and gene model are required in reads mapping and expression quantification and are downloaded from Omicsoft's reference library website [39]—; and (6) s3cmd command line tool [40] to transfer data to and from S3.

In principle, all required software can be installed on-the-fly when each EC2 instance is launched in the cloud. However, the total time required for system configuration is about 50 minutes if starting from the default Amazon's Linux Amazon Machine Image (AMI) 2013.03 (ami-3275ee5b). A better solution is to create a customized AMI with all the needed tools preinstalled and configured. This AMI is then used as a template for launching new EC2 instances. This approach saves 50 minutes when launching a new EC2 instance from this customized AMI, which adds up quickly when several hundred EC2 instances are launched in the cloud. Another advantage to using this customized AMI is reliability; all the needed software packages are properly configured and tested, which eliminates mistakes in system configuration. Stormbow is distributed with a script file that contains all the Unix shell commands needed to create this AMI.

### 2.4. *RNASeq.sh*: A Script for Analysing a Single Sample

When an EC2 instance is launched from the customized AMI above, it comes with all required software. Although it is ready for processing RNA-Seq data, it does not know which sample to process and/or how to analyse it. These two problems are solved through the *cloud-init* mechanism [41], which passes a piece of user-data to an EC2 instance when it is launched. The user-data itself is an executable shell script that is responsible for the following: (1) setting the proper environment variables, such as SAMPLE, GENOME, GENE_MODEL, and REFERENCE_LIBRARY and (2) downloading and executing *RNASeq.sh*, which contains step-by-step data processing instructions. The separation of *RNASeq.sh* from the customized AMI allows for flexibility and eases maintenance of the Stormbow pipeline. Software packages and reference genomes do not change very often, but RNA-Seq datasets and their analyses might vary with platforms and projects. *RNASeq.sh* is fetched on-the-fly by each EC2 instance, and tailored RNA-Seq analysis can be achieved by customizing *RNASeq.sh*.

As shown in the right side of Figure 1, the main tasks for the default *RNASeq.sh* script are as follows: (1) fetching sequence data from S3; (2) mapping reads to the reference genome; (3) quantifying gene and transcript expression; (4) quality checking of mapped reads; and (5) uploading result files to S3. The script also keeps track of the progress and status of the application's execution and collects and records runtime metrics, such as processing time, transfer time, and file sizes. In addition, *RNASeq.sh* handles some common and foreseeable errors to make the workflow more robust. For instance, it is not uncommon for a data transfer from S3 to EC2 to fail due to network congestion. Our script handles this failure by waiting a few minutes and attempting to download the data again instead of failing and terminating the instance. When the analysis is finished, this EC2 instance is terminated immediately to release all the requested resources.

### 2.5. *Stormbow.pl*: A Script to Launch Multiple EC2 Instances in the Cloud

So far, we have explained all the details on how to analyse a single sample in the cloud, including the hardware, software, and the analysis workflow. Now, it is time to discuss utilizing multiple EC2 instances to process many data files in parallel. Stormbow includes *Stormbow.pl*, a Perl script that can be run from any Linux computer with an internet connection to start EC2 instances in the cloud. *Stormbow.pl* takes a manifest file (to be discussed below) as input, prepares a piece of user-data for each sample listed in the manifest file, and writes this user-data to local files. The user-data files are shell scripts containing instructions for the EC2 nodes on how to process each specific sample. As a consequence, all EC2 instances process different samples in parallel, which significantly reduces the clock time of RNA-Seq data analysis. Amazon offers virtually unlimited CPU resources, and thus provides a computational environment that is ideally suited to large-scale RNA-Seq data analyses. As a result, Stormbow can easily scale up or down with the number of RNA samples, and the total running time remains almost constant no matter how large an RNA-Seq study is.

### 2.6. Manifest File and Consistent Naming Convention for Ease of Use

Another problem occurs when many EC2 instances are launched to process data in parallel. Each EC2 instance generates multiple result files that have to be uploaded to S3. The default file names might be too generic or even exactly the same. If we simply upload them to S3, we risk name conflicts and the overwriting of result files. For an RNA-Seq study with several hundred subjects, there will be several thousand result files generated when using OSA to analyse them. All these files need to be named consistently and managed properly.

In order to centralize and manage different types of results files in S3 in an automated fashion, a manifest file and naming convention are introduced. The manifest file is a plain text file to describe the subjects in an RNA-Seq project. Each subject has a corresponding entry in the manifest file, and each entry follows the convention below:

 
*J1* s3://bt/J1_1.fq;s3://bt/J1_2.fq s3://bt/out
 
*J2* s3://bt/J2_1.fq;s3://bt/J2_2.fq s3://bt/out

Each entry consists of three fields separated by spaces or tabs: (1) a unique identifier; (2) raw sequence data in S3; and (3) an output location in S3. The naming convention, together with the unique identifier for each subject, controls how all the intermediate and result files are named, where these files are stored, and how they are logically organized in S3. For instance, the gene count table for sample *J1* has a file name *J1.Gene.Count.txt*, while its transcript count table is accordingly named as *J1.Transcript.Count.txt*. The manifest file and naming convention work together to guarantee all files are named and stored consistently and to avoid overwriting any file associated with another subject. 

### 2.7. Merging of Results for Individual Samples and the Generation of the Gene Count Table

A Perl script, *Merge.pl*, was written to automate the merging of results. The script first fetches the result files corresponding to each subject from S3 using the s3cmd command line tool. It then calls *Merge.R*, an R script to perform the joining, and then puts the merged results back in S3. In addition to the generation of a combined gene and transcript count table, the alignment and mapping summary statistics for each sample are also merged. The gene count table can be directly fed as input to differential expression analysis packages such as DESeq or edgeR. 

## 3. Results and Discussions

### 3.1. A Practical Test Runs of Stormbow

We applied Stormbow to analyse those 178 RNA-Seq subjects listed in Supplementary Table S1 which is available online at http://dx.doi.org/10.1155/2013/481545. All 178 subjects are pair-ended sequenced by Illumina HiSeq 2000 platforms. The practical steps are described as follows. We first made copies of the dataset for the original subjects onto an encrypted 2 TB hard drive and shipped it to Amazon via FedEx. Amazon then copied the data to our specified S3 bucket. This took 2 days, including FedEx shipping time. After the sequence data was available in S3, we created a manifest file, in which each sample was given a unique identifier, locations of inputs, and an output folder in S3. Then, we ran the *Stormbow.pl* script to launch 178 m1.xlarge instances in the cloud. It took an average of 7 hours to process a sample, including sequence download, analysis, and upload of result files. After the analysis of 178 samples was completed, we merged the results and shipped an empty hard drive to Amazon for encrypted data export, which took another 5 days including hard drive shipment time to and from Amazon. Lastly, the large amount of data in S3 was deleted to eliminate continuing charges for data storage.

In total, the RNA-Seq analysis of 178 samples using Stormbow in the cloud was done in less than 10 days, and the total cost was less than $1,000, including Amazon Import and Export charges. Amazon charges $0.50 per hour for m1.xlarge instances, which made the average EC2 cost of processing a single sample $3.50. The total EC2 cost for 178 samples was $650. For the Amazon Import service, the total cost was ~$150 for a single 2 TB hard drive, including (a) $30 for FedEx shipping, (b) a flat $80 charge per device, and (c) $42 for the data loading fee, which is calculated at $2.49 per data-loading hour. The cost for the Amazon Export service was the same—~$150 in our analysis.

**Figure 2:**
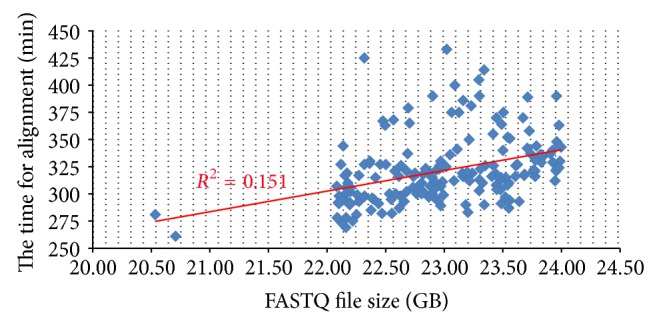
The relationship between the alignment time and the FASTQ file size. Surprisingly, the running times for those samples with a comparable number of sequence reads varyes significantly. The properties of reads, such as indels, mismatches, and multiple mapping, contribute to the difference in running time. More time fluctuations result from the performance difference among EC2 instances.

The running metrics are reported in supplementary Table S1. The time required for analysis varies from sample to sample, but the majority of samples were processed within 6 to 8 hours. On average, each sample has 53 million pairs of reads, and the average FASTQ file size is 23 GB. The average download and upload times are 18 and 12 minutes, respectively. To create a gene count table from a BAM alignment file it takes an average of 24 minutes, and the same time is needed to create a count table at transcript level. The mapping of more than 100 M reads to a reference genome is the most time consuming step and takes an average of 321 minutes to accomplish. The relationship between the alignment time and the FASTQ file size is shown in Figure 2. There is no discernibly linear relationship between the computational time and the number of sequences.

We are puzzled by the trend shown in Figure 2. Each sample is analyzed in identical environments, including CPU, software, and parameters for alignment, but the running times seem to vary significantly for samples with comparable numbers of reads. After discussing our results with OSA developers and rerunning the analysis for sample *J1* four times in the cloud, we discovered two main reasons to explain the observed difference. First, the time to map a single sequence read to a reference genome varies significantly. The properties of reads have a dramatic effect on running time. Reads that are mapped with indels and mismatches require longer alignment time. Also, samples with more unmapped reads and multiple mapped reads will require additional alignment time. Moreover, sequence quality impacts alignment time as well. The total alignment is not simply determined by the number of sequence reads. Secondly, not all EC2 instances are identical in terms of performance. If we repeat the analysis in a local environment, the running times are nearly identical. However, when we analysed sample *J1* in the cloud 4 times using the same computational environment, the running times were 5 h:20 m, 5 h:06 m, 5 h:19 m, and 5 h:44 m. When alignment begins, all the sequence reads and reference genomes are in local instance storage drives, so we cannot attribute the difference to network fluctuations. Therefore, we can assume that the EC2 instances themselves give rise to the observed time difference. An EC2 instance is a virtual machine, and many EC2 instances can run on a single piece of hardware. Depending on the neighbouring EC2 virtual machines, each EC2 instance may get slightly varying amounts of perceived CPU, memory, and I/O bandwidth. As a consequence, the analysis time does vary. Despite the running time difference, the result files are identical for all separate runs of the same sample. 

### 3.2. Amazon Cloud as an Execution Environment for Stormbow

When we implemented Stormbow, we chose Amazon's cloud as the execution environment over local clusters for two reasons: (1) Amazon offers virtually unlimited storage and CPU resources for easy and fast scaling to handle large RNA-Seq studies; the option to set up a local high-performance cluster to keep pace with the computational and storage challenges for NGS data analysis was not a feasible option for us; and (2) Amazon provides on-demand access to a wide range of cloud infrastructure services, charging you only for the resources you actually request. This “pay-as-you-go” model works better for our research because we do not need dedicated resources for RNA-Seq data analysis year-around. With its massive economies of scale, Amazon is continually driving cost down and reducing costs to the end users. Furthermore, cloud computing offers operational advantages, such as setting up infrastructure in minutes rather than months, and completing massive computational tasks with a large number of resources quickly. 

We chose S3 to centralize data storage, including inputs, intermediate results, and outputs from every computational step in Stormbow. S3 provides virtually unlimited storage space for cloud computing and stores multiple copies of its data, which guarantee the safety of data in Amazon's cloud. All objects in S3 have unique identifiers and can be fetched in parallel without input/output (I/O) congestion. This parallelism is critical when multiple EC2 instances are uploading or downloading large amounts of data simultaneously to and from S3. 

### 3.3. The Choice of OSA for Reads Mapping and Expression Quantification

To apply RNA-Seq to differential expression analysis, we need first to quantify the gene expression level in each sample, and then run statistical tests to identify DE genes. The quantification task typically involves (1) the mapping of large number of reads to a reference genome or transcriptome, and (2) the estimation of gene and isoform abundances based on the read mappings. In addition to OSA, there are other algorithms available to map reads in RNA-Seq studies, such as Tophat [36]. The reasons we chose OSA are as follows: firstly, OSA is several times faster than Tophat with better sensitivity and less false positives; secondly, OSA takes a parameter file to control how the reads are mapped to a reference genome or transcriptome, which is more versatile to pass to an EC2 node in the cloud than using a command line parameter; thirdly, the vendor of OSA has provided and maintained prebuilt genome and gene models for many common organisms, which removes the time and complexity of developing these by ourselves; finally, OSA not only is an alignment tool, but also has a built-in subcommand for expression quantification. Of course, Tophat remains a tool widely used in the RNA-Seq community. In fact, we use it for the discovery of novel genes and isoform or the detection of gene fusion. However, for differential studies in which we investigate the effects of compound treatment and/or gene knockdown, our main interest is to identify those known genes that are differentially expressed, not to discover novel genes or transcripts. In this scenario, OSA fits our needs better. 

To generate a gene count table from mapped reads is complex. The first major complication in quantification is the fact that RNA-Seq reads do not always map uniquely to a single gene or isoform. The transcripts from which RNA-Seq reads are derived are not always uniquely determined. Paralogous gene families, low-complexity sequence, and high sequence similarity between alternatively spliced isoforms of the same gene are primary factors contributing to mapping uncertainty. In addition, polymorphisms, reference sequence errors, and sequencing errors require mismatches and indels to be allowed in read alignments and further contribute to lower confidence in mappings. As a consequence, a significant number of reads are multireads: reads that have high-scoring alignments to multiple positions in a reference genome or transcript set. Another challenge in quantification is gene overlapping. Some regions are shared by more than one gene. If a read is mapped to such regions, to which gene this read should be counted is ambiguous. OSA implements an algorithm similar to RSEM [42] to deal with gene overlapping and multireads. OSA estimates maximum likelihood (ML) expression levels using an expectation maximization (EM) algorithm and gives more accurate gene expression estimates than those using only unique reads or other alterative rescue strategies.

### 3.4. Stormbow versus Rainbow and Myrna

Both Rainbow [32] and Stormbow are cloud-based tools we have developed for large-scale sequence data analysis, but they have different goals. Rainbow is developed for whole genome sequence data analysis, where typically there are more than 1 billion reads per sample. With Rainbow, instead of launching a single EC2 instance to map billions of reads, a compute cluster is launched in the cloud to process the data in parallel with the output being all the Single Nucleotide Polymorphisms (SNP) for a sample. In contrast, Stormbow is designed for much smaller RNA-Seq data analysis, and its output is a gene count table to represent the expression levels of all genes in a gene model.

Myrna [25, 43] is another cloud computing tool for calculating differential gene expressions in large RNA-Seq datasets. Myrna was developed more than 3 years ago, and it uses a modified Bowtie [44] for short read alignment. The Bowtie version in Myrna does not handle insertion and deletion when mapping reads to a reference genome. Thus, Myrna's biggest drawback is that it does not attempt to align reads across junctions, assemble isoforms, or otherwise analyse on the isoform or junction level. Unfortunately, many sequence reads from RNA-Seq indeed span two or more exons. Although Stormbow and Myrna are both designed to process large RNA-Seq datasets, the parallelism is achieved by different strategies. Stormbow launches multiple individual EC2 instances to process RNA-Seq samples in parallel, while Myrna launches a large compute cluster to sequentially process RNA-Seq data sample by sample, and parallelize data analysis within each RNA-Seq run. Of course, mapping millions of reads can be easily accomplished in parallel, but not all steps in data analysis can be easily parallelized. Compared to Myrna, Stormbow achieves a higher parallelism than that achieved by Myrna and virtually can scale up to a large RNA-Seq study of any size.

### 3.5. The Advantages of Stormbow

RNA-Seq is a powerful technology that is predicted to replace microarrays for transcriptome profiling [1]. The amount of data coming out of an RNA-Seq experiment can be staggering, orders of magnitude more than microarrays. For instance, a typical raw CEL data file generated from Affymetrix HT HG-U133+ PM array is 5 MB per sample, whereas RNA-Seq data is about 23 GB in our study. The raw data alone increases 4,600-fold. Leveraging recent advances in sequencing technology, RNA-Seq experiments produce larger amounts of sequence data at lower costs, and quite often by small to midsize research groups that have no access to local high-performance computing environment. The cloud-based Stormbow processes the large volume of RNA-Seq data quickly at an affordable price as our in-house test run has demonstrated. 

Stormbow is fully scalable and developed for large-scale RNA-Seq differential studies. It is written using Perl, Shell, and R scripts, and it is simple to be deployed and used by third parties. It can be used out of the box to process Illumina RNA-Seq datasets. The manifest file is the only file that a user needs to prepare in order to run Stormbow. In essence, Stormbow is a wrapper of OSA, but it hides the complexity of directly using OSA for RNA-Seq data analysis in the cloud. More importantly, the performance of Stormbow has been tested by practically applying it to analyze 178 RNA-Seq samples simultaneously. 

### 3.6. Lessons Learned with Amazon Cloud Computing

**Figure 3:**
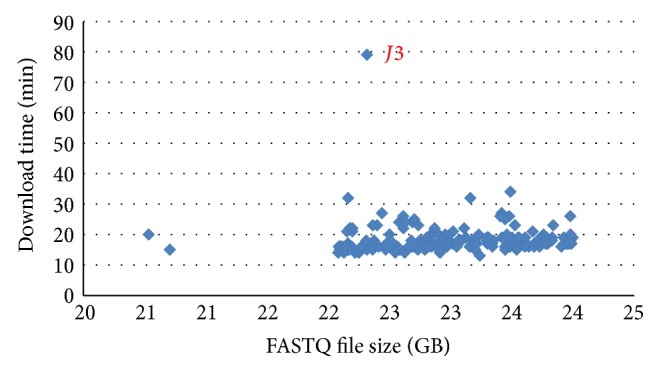
The relationship between the download time and the FASTQ file size. The average FASTQ file size is 23 GB, and the download time is 18.4 minutes on average. For sample *J3*, it takes 79 minutes to download 22.3 GB.

To analyse large volumes of data in Amazon's cloud is different from in a local environment. Quite often, large datasets are stored in S3. They have to be downloaded from S3 to EC2 instances first, which is not trivial. Physically, the data transfer is done through Amazon's internal network, which is not always reliable. Network congestion or failure is not uncommon for. We encountered this situation a few times during the development of Stormbow and in our test run. As shown in Figure 3, the download time was an average of 18.4 minutes per sample; however, it had taken as long as 79 minutes to download Sample *J3* in our test run of Stormbow. We repeated the analysis for *J3*, and the download time was about 18 minutes. Clearly, the network congestion can cause nontrivial download delays. In Stormbow, the s3cmd command line tool is used to fetch sequence data from S3 to an EC2 instance. When the network connection is lost during an upload or download, s3cmd responds with an error. In our test run of those 178 samples, we received this type of error twice. When you move data around in the cloud, it is always advised to check and make sure the data transfer is successful regardless of the data size.

Amazon offers a variety of EC2 instances for you to choose with different pricing models. You need to understand how your algorithm works and benchmark to choose the best option. In Stormbow, the m1.xlarge instance was chosen to run OSA due to reasons stated previously. When we tested on an instance type with more CPU cores, the running time was not strikingly different. The reasons behind this are as follows: first, more threads cause I/O congestion since all threads write the results into the same hard drive; second, more threads increase the parallelism only for the first step in OSA, but not the second step. For our application, an instance with higher absolute CPU and memory performance was not a better option.

For those developers who want to wrap other standalone applications and launch multiple EC2 instances to run them in parallel, they might be facing some common challenges, including the following: (1) transferring large amounts of data; (2) launching many EC2 instances automatically; (3) instructing instances to perform specific computations; and (4) managing the large number of result files in S3. In Stormbow, we wrote a driver script to automate the launch of EC2 instances, and made use of the cloud-init mechanism to pass instructions to new EC2 instances. The manifest file and naming convention standardize how the intermediate and result files are named and logically organized in S3. All these ideas and practices have proven to work well in Stormbow, and can be helpful for developing other cloud-based bioinformatics tools.

## 4. Conclusions

In this paper, we described the motivation for the development of Stormbow, its implementation, and evaluation. Stormbow is a cloud-based software package that wraps OSA for parallel execution in the cloud. It is designed for large-scale RNA-Seq studies in which several hundred or even thousand samples are sequenced for differential analysis. It is cost effective and simple to use as we have demonstrated in our test run. Stormbow is open-source based and implemented using Perl, Shell, and R scripts. The source code for Stormbow is freely available for download from the Stormbow website [45]. OSA is free to academic users, but for commercial users, you need to contact Omicsoft Inc. Since all computations are performed in the Amazon cloud, a user will need to pay Amazon to be able to run analyses.

## Supplementary Material

Descriptions on 178 RNA-Seq samples and their detail running matrices, including (1) the number of pair-ended reads, (2) the original FASTQ file size, (3) the BAM file size after alignment, (4) running times for download of FASTQ files from S3 to EC2, alignment of reads to reference genome, quantification of gene and transcript expressions, upload of results onto S3, and the total running time.
